# Effects of pharmacological inhibition of the sodium‐dependent phosphate cotransporter 2b (NPT2b) on intestinal phosphate absorption in mouse and rat models

**DOI:** 10.1002/prp2.938

**Published:** 2022-02-22

**Authors:** Xiaojun Wang, Yanping Xu, Xiaohong Yu, Asim Dey, Hong Y. Zhang, Charity M. Zink, Derek Wodka, Gina Porter, William F. Matter, Leah Porras, Charles A. Reidy, Jeffrey A. Peterson, Brian E. Mattioni, Joseph V. Haas, Mark C. Kowala, John R. Wetterau

**Affiliations:** ^1^ Lilly Research Laboratories Eli Lilly and Company Indianapolis Indiana USA

**Keywords:** NPT2b, Phosphate metabolism

## Abstract

An excess phosphate burden in renal disease has pathological consequences for bone, kidney, and heart. Therapies to decrease intestinal phosphate absorption have been used to address the problem, but with limited success. Here, we describe the in vivo effects of a novel potent inhibitor of the intestinal sodium‐dependent phosphate cotransporter NPT2b, LY3358966. Following treatment with LY3358966, phosphate uptake into plasma 15 min following an oral dose of radiolabeled phosphate was decreased 74% and 22% in mice and rats, respectively, indicating NPT2b plays a much more dominant role in mice than rats. Following the treatment with LY3358966 and radiolabeled phosphate, mouse feces were collected for 48 h to determine the ability of LY3358966 to inhibit phosphate absorption. Compared to vehicle‐treated animals, there was a significant increase in radiolabeled phosphate recovered in feces (8.6% of the dose, *p* < .0001). Similar studies performed in rats also increased phosphate recovered in feces (5.3% of the dose, *p* < .05). When used in combination with the phosphate binder sevelamer in rats, there was a further small, but not significant, increase in fecal phosphate. In conclusion, LY3358966 revealed a more prominent role for NPT2b on acute intestinal phosphate uptake into plasma in mice than rats. However, the modest effects on total intestinal phosphate absorption observed in mice and rats with LY3359866 when used alone or in combination with sevelamer highlights the challenge to identify new more effective therapeutic targets and/or drug combinations to treat the phosphate burden in patients with renal disease.

## Graphical abstract

NPT2 inhibition, effect on intestinal phosphate absorption.

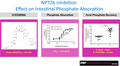

**Abbreviations**
F: bioavailability
NHE3: sodium hydrogen exchanger 3
NPT2a: sodium‐dependent phosphate cotransporter 2a
NPT2b: sodium‐dependent phosphate cotransporter 2b
NPT2c: sodium‐dependent phosphate cotransporter 2c
PiT1: phosphate transporter 1
PiT2: phosphate transporter 2

## 1 INTRODUCTION

In healthy adults, the amount of dietary phosphate absorbed is balanced by phosphate excreted through the kidney.^1^ In chronic kidney disease (CKD) patients, the ability to excrete phosphate through the kidney is compromised and several compensatory mechanisms are activated to help maintain serum phosphate levels within the normal range.^2^ These include an elevation in FGF23 and PTH, which decrease the reabsorption of filtered phosphate in the kidneys, and a reduction in 1,25‐dihydroxyvitamin D, which decreases intestinal NPT2b expression and phosphate absorption. However, in advanced CKD and end‐stage renal disease (ESRD), the compensatory mechanisms are inadequate, and hyperphosphatemia ensues.^3^ Although compensatory mechanisms help attenuate the rise in serum phosphate levels, they along with the increased phosphate burden have detrimental effects on bone, kidney, and heart.^2^, ^4^ If one was able to adequately decrease dietary phosphate absorption, this pathological sequence of events may be minimized.

To balance intestinal phosphate absorption with the decrease in renal excretion in CKD and ESRD, dietary phosphate restriction and phosphate binder therapies have been used. Given the high levels of phosphate in Western diets and typical poor compliance encountered with restrictive diets, controlling phosphate by dietary phosphate restriction can be challenging.^5^, ^6^ In addition, phosphate binders have limited phosphate binding capacity, which limits their efficacy.^7^ Based upon decreases in urinary phosphate or increases in fecal phosphate content with treatment, phosphate binders appear to only inhibit phosphate absorption around 20%–40% for a normal diet.^8^, ^9^, ^10^, ^11^ This may explain their limited ability to control FGF23 and PTH levels in CKD.^8^, ^12^, ^13^, ^14^ A small study with severe dietary phosphate restriction that achieved a 50% decrease in urinary phosphate produced a profound 70% decrease in PTH in humans,^15^ highlighting the potential for a new therapy that robustly decreases dietary phosphate absorption as a monotherapy or in combination therapy.

The inability to control phosphate levels in CKD and ESRD has led to a search for more effective therapies to limit dietary phosphate absorption. In the human intestine, dietary phosphate is absorbed through active transport and passive diffusion.^16^ Current therapies used to limit dietary phosphate absorption decrease the free phosphate concentration in the intestinal lumen, suggesting they would decrease absorption through an effect on passive diffusion. Recently, the NHE3 inhibitor, tenapanor, was shown to inhibit intestinal phosphate absorption by blocking the paracellular diffusional pathway.^17^, ^18^

The need for more effective therapies has led to a search for inhibitors of active phosphate transport. Based upon preclinical animal models,^19^, ^20^ the intestinal high‐affinity sodium‐dependent phosphate cotransporter 2b (NPT2b) with a $K_{m}^{P_{i}}$ = 10 µM^21^ is thought to play a prominent role in intestinal phosphate active transport. At the millimolar phosphate concentrations found in the human intestine after a meal,^16^ NPT2b should transport phosphate at its maximal velocity. More recently, studies in rats and humans have implicated the ubiquitously expressed, low‐affinity sodium‐dependent phosphate transporters PiT1 and possibly PiT2 in intestinal phosphate absorption.^22^, ^23^

The contribution of passive diffusional transport and the various sodium‐dependent active transporters in phosphate absorption is uncertain in both preclinical models and humans. Complicating our understanding, their contributions undoubtedly vary under different conditions, for example, the concentration of free phosphate in the intestine, the age of the animals or individuals, their health status (healthy, CKD or ESRD), the 1,25‐dihydroxyvitamin D status (upregulates active transport and NPT2b^24^, ^25^), and counter‐regulation of other pathways in response to therapy. As it is unlikely any one therapy will effectively control the phosphate burden in the more challenging patients, it is also important to understand how different therapies work in combination.

To further address these issues, a medicinal chemistry campaign was used to identify a potent NPT2b inhibitor, LY3358966. The compound was used to explore the role of NPT2b‐mediated active transport in phosphate absorption in two preclinical animal models under carefully controlled conditions. In addition, the ability of the inhibitor to work in combination with the phosphate binder, sevelamer, was also investigated.

## 2 MATERIALS AND METHODS

### 2.1 In vitro activity

T‐Rex^™^ Chinese Hamster Ovary cells (CHO; Thermo Fisher Scientific) stably expressing human, mouse, or rat NPT2b under a tetracycline‐inducible promoter were generated using standard cDNA transfection and clone selection procedures (see Supporting Information). Cells were plated in 96‐well CytoStar‐T^®^ scintillating microplates and were incubated overnight in growth media plus 100 ng/ml of tetracycline to induce expression of NPT2b. The next day, cells were washed 3 times with 200 µl assay buffer (137 mM NaCl, 5.4 mM KCl, 2.8 mM CaCl_2_, 1.2 mM MgSO_4_, and 14 mM Tris‐HCl buffer, pH 7.5). LY3358966 serially diluted 1‐to‐3 in DMSO was added to cells with assay buffer. An equal volume of H_3_^33^PO_4_ (PerkinElmer) in assay buffer supplemented with 5 µM potassium phosphate was then added to initiate radiolabeled phosphate (^33^P‐phosphate) uptake. The final phosphate concentration was 2.5 µM and the final DMSO concentration was 1%. Following a 60‐min incubation at room temperature, an equal volume of assay buffer containing 400 µM phloretin was added to stop ^33^P‐phosphate uptake. The plate was immediately read on a Wallac MicroBeta Trilux liquid scintillation counter. Percent inhibition at all concentrations tested was calculated, and IC_50_ values were then determined using a 4‐parameter logistic curve fitting equation.

Similar methods were used to measure the effects of LY3358966 on other transporters of interest, except that constitutive promoters were used to generate CHO cells over‐expressing human PiT1 and PiT2. The ^33^P‐phosphate uptake was measured after 45 min at 20°C for the NPT2a, NPT2c, PiT1, and PiT2 assays.

### 2.2 LY3358966 synthesis

The detailed synthesis of LY3358966 (4‐[2‐[2,6‐difluoro‐4‐[[2‐[[3‐[[4‐(4‐hydroxybutylcarbamoyl)‐2,2‐dimethyl‐piperazin‐1‐yl]methyl]benzoyl]amino]‐4,5,6,7‐tetrahydrobenzothiophene‐3‐carbonyl]amino]phenyl]ethyl]benzoic acid) is described in the Supporting Information.

### 2.3 LY3358966 formulation

A spray‐dried solid dispersion (SDD) containing 30% LY3358966 was made by adding the free base of LY3358966 to poly‐1‐vinylpyrrolidone‐co‐vinyl acetate (PVP‐VA) in methanol. Two mole equivalents of NaOH were added to the slurry, which was then bath sonicated until a clear yellow solution was formed. The solution was slowly pumped into a spray dryer with a stream of hot nitrogen, resulting in a solid powder that was collected and further dried in a vacuum oven at 50°C. In one study, the solid formulation (LY3358966 SDD) was placed in a capsule, while in other studies, it was dissolved in water before dosing.

### 2.4 In vivo studies

All animal procedures were approved by and conducted in accordance with the Eli Lilly and Covance Institutional Animal Care and Use Committee guidelines. Mice and rats were fed Teklad Global 14% protein rodent maintenance diet (Envigo) containing 0.6% of phosphorus (0.3% non‐phytate phosphorus).

### 2.5 Acute phosphate uptake into plasma

Male C57BL/6 male mice (*n* = 7–9/group) at the age of about 8–9 weeks (21–29 g) were fasted overnight and throughout the study. Mice were dosed orally with varying doses of LY3358966 SDD dissolved in water or a PVP‐VA vehicle control. Fifteen minutes later, a radioactive phosphate dosing solution (200 µl of 16.25 mM Na_2_HPO_4_, 0.9% saline, pH 7.4 supplemented with about one million dpm of H_3_^33^PO_4_) was given by oral gavage. This dose of phosphate was chosen because it approximates the mouse equivalent of the amount of bioavailable phosphate in a human meal which is 4 mg/kg phosphorus, or 320 mg phosphorus for an 80 kg person on a phosphate restricted diet. Fifteen minutes later, blood was collected and radioactivity in the plasma was determined by scintillation counting. The inhibition of acute phosphate uptake into plasma by LY3358966 was determined by comparing the counts in the plasma from LY3358966‐treated animals to that in the plasma of the vehicle‐treated animals. ED_50_ values were then determined with a 4‐parameter logistic curve fitting model using GraphPad Prism 7.04.

To test the ability of LY3358966 to inhibit intestinal NPT2b activity in mice over time, the above mice were bled again at 3 h post compound dose to determine the residual radioactivity in the plasma. At 4 h post compound dose, the animals were again dosed with phosphate using threefold more H_3_^33^PO_4_ than used in the first oral dose. Fifteen minutes later, blood was collected and radioactivity in the plasma was used to calculate LY3358966 inhibition of phosphate uptake into plasma. For this calculation, the residual counts at 3 h were first subtracted from the final counts at 4 h. The 3‐h counts were typically <15% of the 4‐h counts.

A similar method was used to determine the ability of LY3358966 to inhibit acute phosphate uptake into plasma in Sprague Dawley rats (192–225 g, *n* = 14/group). In the rat studies, a 2 ml volume of the radioactive phosphate dosing solution (about 10 million dpm of H_3_^33^PO_4_) was dosed to each animal. Due to the low inhibitory activity at 15 min after dosing the compound, the effect at 4 h was not determined.

To test the ability of a solid dose formulation of LY3358966 to inhibit phosphate absorption, LY3358966 SDD was incorporated into a capsule (Torpac^®^ capsule dosing kit) at an amount that provided a 1.8 mg/kg dose of active pharmaceutical ingredient (API). LY3358966 SDD or PVP‐VA as placebo, either in capsules or dissolved in water, were dosed to mice. Forty‐five minutes later, they were dosed with radiolabeled phosphate, and the ability of LY3358966 to inhibit acute phosphate uptake, compared to its placebo control, was determined as described above. A 45‐min time point was utilized to account for the added time required for the capsule to dissolve in the stomach.

### 2.6 Gastric emptying assay in mice

Male C57BL/6 mice, approximately 7–8 weeks old (18–21 g), were fasted overnight. Then, the mice were orally administered a single dose of vehicle (water) or varying doses of LY3358966 SDD dissolved in water. Fifteen minutes later, radiolabeled phosphate was given by oral gavage. Another 15 min later, the animals were sacrificed, and intact stomachs were collected and placed in 50‐ml conical tubes. Ten milliliter of 1 N NaOH was added to each tube and the stomachs were digested at 37°C overnight. Radioactivity in 100 µl digested stomach homogenate was quantitated by scintillation counting. The percent radiolabeled phosphate recovered in the stomach was calculated as the percentage of dpm recovered in the stomach versus the total dpm of radiolabeled phosphate administered.

### 2.7 Inhibition of phosphate absorption in mice

Male C57BL/6 male mice, 9 weeks old (23–28 g, *n* = 10/group), were dosed with either placebo or LY3358966 SDD (9 mg/kg API) dissolved in water at *t* = −4.25, −0.25, and 3.75 h. The animals were fasted from *t* = −4 to *t* = 0 h. At *t* = 0, the animals were dosed with radiolabeled phosphate as described above and were given free access to food. Feces were collected at 6, 24, and 48 h, digested overnight at 37°C in 1 N NaOH, and the recovered radioactivity was determined. Most of the radiolabeled phosphate was recovered in the first 24 h. In a control study, we demonstrated that 82 ± 1.34% (mean ± *SEM*, *n* = 10) of a non‐absorbed control, ^14^C‐polyethylene glycol‐4000 (PerkinElmer), could be recovered in the feces by 48 h. In all cases, the investigators collecting feces were blinded to the treatment.

### 2.8 Inhibition of phosphate absorption by LY3358966 alone or in combination with sevelamer HCl in rats

Male Sprague Dawley rats, 260–287 g for the first study and 274–362 g for the second study, were fasted for 4 h (the first 4 h of the light cycle) then dosed with placebo, a 50 or 150 mg/kg sevelamer HCl water dispersion, LY3358966 SDD (10 mg/kg API) dissolved in water, or a combination of LY3358966 SDD and sevelamer HCl. Fifteen minutes later, the animals were dosed with radiolabeled phosphate as described above. In the first study, the animals were given free access to food, then feces were collected at 6, 24, and 48 h post radiolabeled phosphate dose. In the second study, the animals were fasted an additional 4 h, then feces, stomach, small intestine, and large intestine were individually collected. The gastrointestinal tract tissues and feces were digested overnight at 37°C in 1 N NaOH, and the recovered radioactivity was determined.

### 2.9 Pharmacokinetic studies

Male CD‐1 male mice were orally dosed with 1, 3, or 10 mg/kg API of LY3358966 SDD dissolved in water or were IV dosed with 1 mg/kg LY3358966 formulated in 25% dimethylacetamide, 15% ethanol, 10% propylene glycol, 25% 2‐pyrrolidone, and 25% water. Each dose group contained six animals. Three animals were bled at 0.25, 0.5, 1, 2, 4, 8, 12, and 24 h, with each animal being bled at alternate time points. Plasma drug levels were determined by HPLC/MS. The unbound fraction of LY3358966 in plasma was determined with equilibrium dialysis and LC‐MS/MS.

### 2.10 Statistical analyses

An unpaired Student's *t*‐test with two‐tails was utilized for the two‐group comparisons in the capsule study and the mouse fecal study. It was also used for multiple group comparisons in the gastric emptying study to provide a statistical test that optimizes the chance to detect a significant effect. One‐way ANOVA with Dunnett's multiple comparisons was utilized for multiple‐group comparisons in other studies.

## 3 RESULTS

### 3.1 In vitro inhibition of sodium‐dependent cotransporters by LY3358966

**FIGURE 1:**
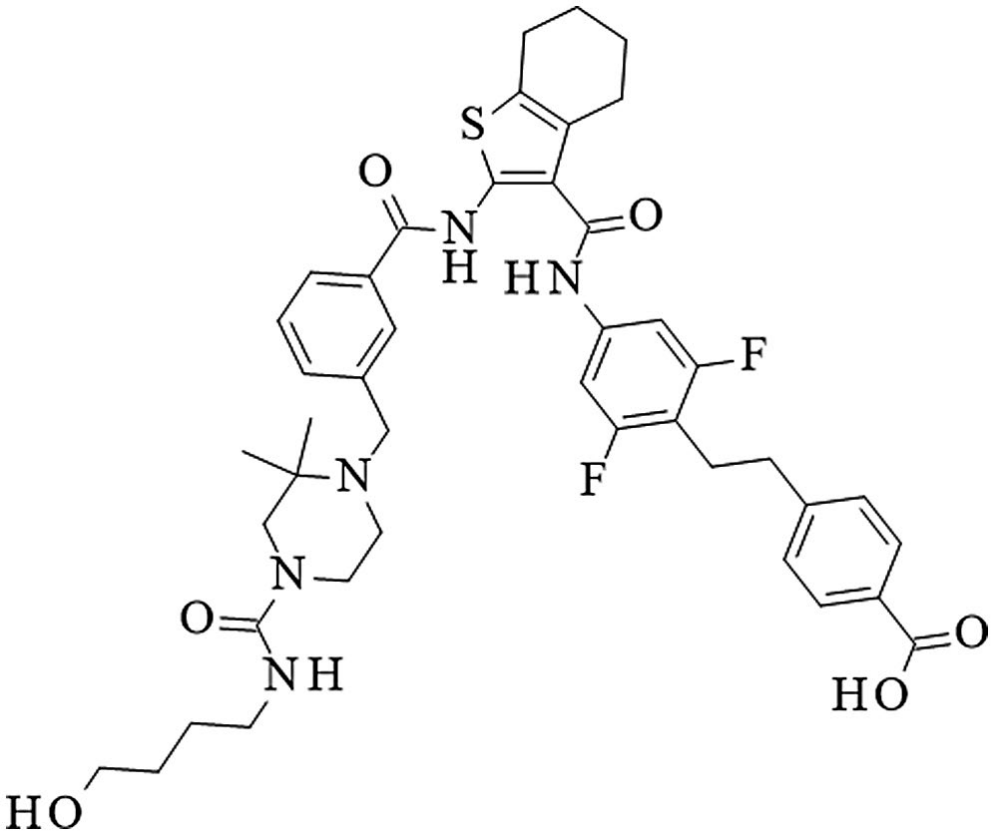
Chemical structure of LY3358966

**FIGURE 2:**
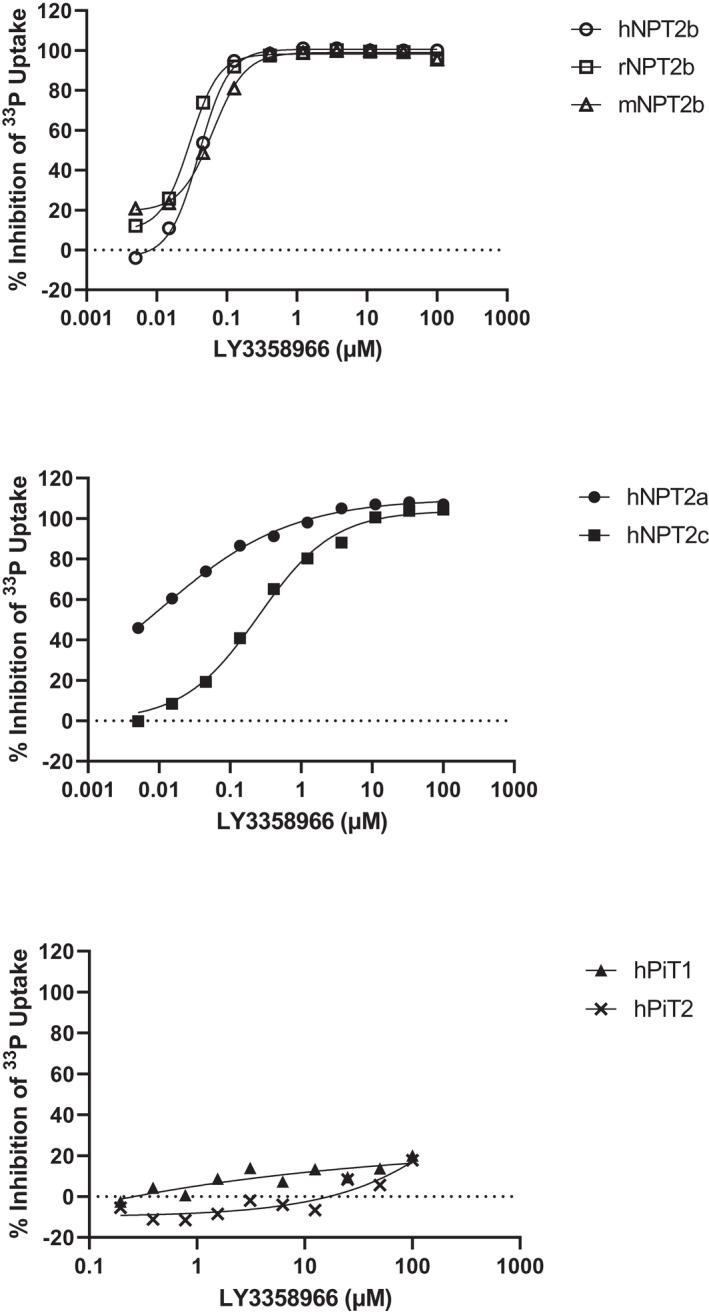
In vitro inhibition of NPT2b, NPT2a NPT2c, Pit‐1, and Pit‐2. Example curves for percent inhibition of phosphate uptake into CHO cells expressing human, rat, or mouse NPT2b (top panel), human NPT2a or NPT2c (middle panel), and human Pit1 or PiT2 (bottom panel) versus no LY3358966. IC_50_ values were determined using a 4‐parameter logistic curve fitting equation and are summarized in Table 1

LY3358966 (Figure 1) was discovered through a medicinal chemistry campaign. It was tested for its ability to inhibit sodium‐dependent ^33^P‐phosphate uptake in CHO cells over‐expressing human NPT2b. LY3358966 inhibited phosphate uptake in a concentration‐dependent manner with an IC_50_ of 32.4 nM (Figure 2; Table 1A). LY3358966 also inhibited mouse and rat NPT2b expressed in CHO cells with similar potencies (Table 1A).

**TABLE 1:** Summary of in vitro inhibition of NPT2b (A) and other human sodium dependent cotransporters (B) by LY3358966

NPT2b assays	IC_50_, nM	*SEM*	*n*
(A)			
Human NPT2b	32.4	13.3	3
Mouse NPT2b	43.9	14.0	3
Rat NPT2b	26.8	8.45	5

Selectivity assays	IC_50_, nM	*SEM*	*n*	Selectivity
(B)				
Human NPT2a	11	1	3	0.3
Human NPT2c	251	10	3	8
Human PiT1	>100 000	N/A	2	>3086
Human PiT2	>100 000	N/A	2	>3086

Note

IC_50_ values represent the geometric mean of individual IC_50_. *SEM* represents the standard error of the geometric mean. *n* represents the number of individual studies on different days. Selectivity represents the ratio of IC_50_ of the selectivity assay to that of the human NPT2b assay.

The in vitro activity of LY3358966 was determined for other members of the human type II sodium phosphate cotransporter family, NPT2a and NPT2c, which are found primarily in the kidney and members of the human type III sodium phosphate cotransporter family, PiT1 and PiT2, which are ubiquitously expressed. LY3358966 was a potent inhibitor of NPT2a and NPT2c (Figure 2; Table 1B). The selectivity of LY3358966 against human NPT2b was 0.3‐fold versus NPT2a and 8‐fold versus NPT2c (IC_50_ of NPT2a or 2c/IC_50_ of NPT2b). The IC_50_ of LY3358966 for inhibition of PiT1 or PiT2 was >100 µM (Figure 2; Table 1B).

### 3.2 Pharmacokinetics of LY3358966 SDD

A SDD of LY3358966 formulated with PVP‐VA (LY3358966 SDD) was dissolved in water and used to determine the pharmacokinetic properties of orally administered LY3358966 in mice. It was administered to mice through an IV route at 1 mg/kg and a P.O. route at 1, 3, and 10 mg/kg LY3358966. The oral bioavailability of LY3358966 was low (9%–12% *F*, Table 2). Systemic exposure in mice following oral dosing was dose‐proportionally increased. LY3358966 is highly protein bound. AUC_unbound_ and *C*_max,unbound_ were estimated to be <2 nM × h and <1 nM, respectively, across all doses.

**TABLE 2:** Summary of LY3358966 pharmacokinetic properties in mice

Dose route, mg/kg	AUC, nM × h	*C*_Max_, nM	*T*_1/2_, h	*T*_MAX_, h	*F*
IV 1	323	N/A	5	N/A	N/A
PO 1	39	25	N/A	0.4	0.12
PO 3	96	50	N/A	0.4	0.10
PO 10	284	83	N/A	0.4	0.09

Note

With an unbound fraction of 0.005 in mouse plasma, plasma AUC of unbound LY3358966 is <2 nM × h and *C*_max_ of unbound LY3358966 is <1 nM across all dose groups.

### 3.3 Effect of LY3358966 SDD on acute phosphate uptake into plasma in mice and rats

**FIGURE 3:**
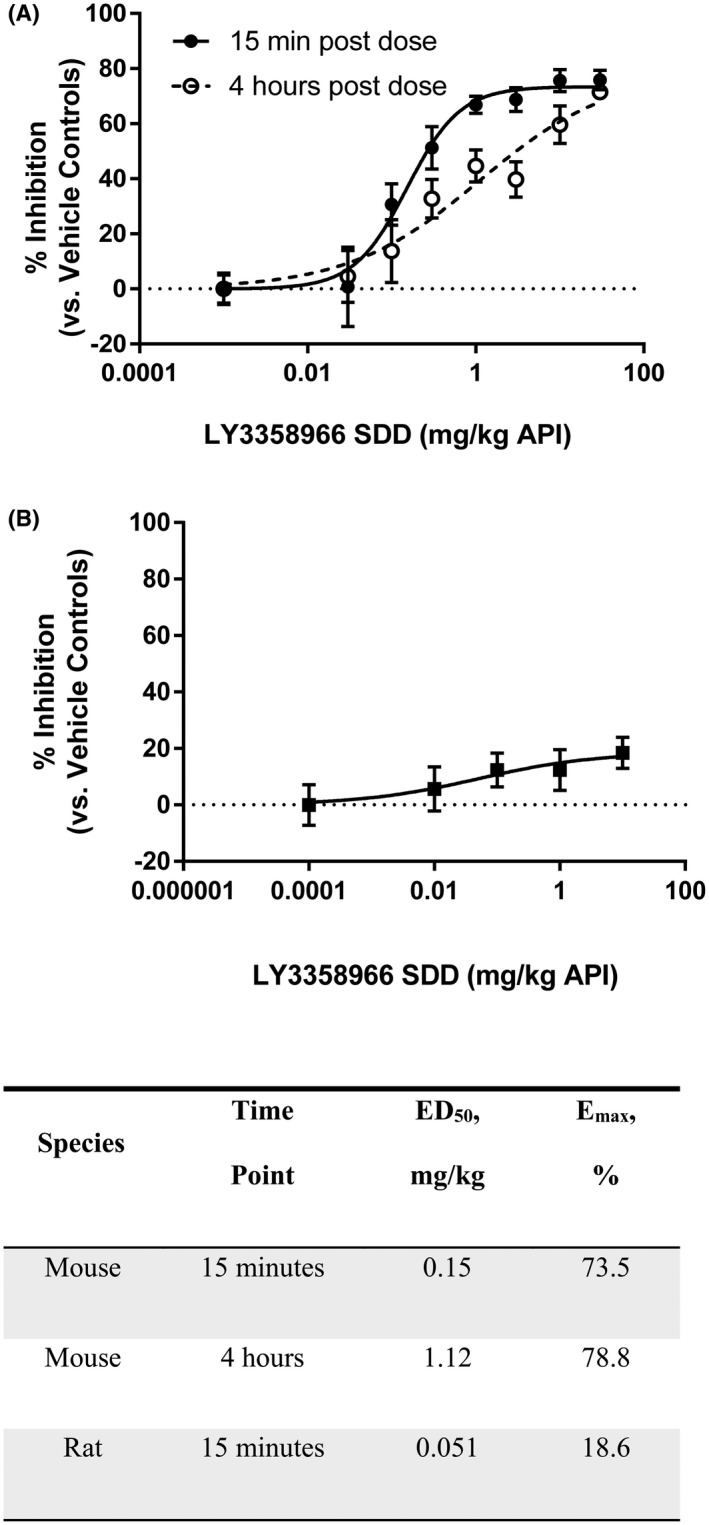
Effect of LY3358966 spray‐dried solid dispersion (SDD) on acute phosphate uptake into plasma in mice (A) and rats (B). Percentage of inhibition was defined as percent decrease of dpm in the plasma of LY3358966 SDD treated groups compared to the average dpm in the plasma of vehicle control groups. The curves were fitted using the 4‐parameter logistic fitting tool GraphPad Prism 7.04. For the purposes of curve fitting, the vehicle was set to a dose of 0.001 or 0.0001 mg/kg. For the placebo control groups, the percent of the total ^33^P‐phosphate dose found in plasma at 15 min was 2.1 ± 0.1% (*n* = 23) in mice and 1.8 ± 0.1% (*n* = 14) in rats (mean ± *SEM*)

LY3358966 SDD was dissolved in water and then orally gavaged to mice at a dose range from 0.03 to 30 mg/kg API. Following an oral dose of ^33^P‐phosphate, LY3358966 dose‐dependently decreased ^33^P‐phosphate appearance in the plasma with an ED_50_ of 0.15 mg/kg measured at 15 min and an ED_50_ of 1.1 mg/kg measured at 4 h post LY3358966 dose (Figure 3A). The calculated maximum inhibition (*E*_max_) of phosphate uptake by LY3358966 at 15 min and 4 h were 74% and 79%, respectively, indicating that active phosphate transport mediated by NPT2b dominates acute phosphate uptake into plasma in mice.

LY3358966 SDD orally gavaged to rats at a dose range from 0.01 to 10 mg/kg API decreased acute phosphate uptake into plasma measured at 15 min post LY3358966 dose with an ED_50_ of 0.051 mg/kg and *E*_max_ of 18.6% in rats (Figure 3B). In two additional studies, a 10 mg/kg dose of LY3358966 decreased phosphate absorption by 28.2% and 19.2%. The mean ± *SEM* for the three studies at 10 mg/kg was 22 ± 3%. In contrast to mice, LY3358966 does not have a robust effect on acute phosphate uptake in rats, suggesting pathways other than NPT2b‐mediated active transport dominate acute phosphate uptake in rats.

### 3.4 Effect of LY3358966 on gastric emptying in mice

**FIGURE 4:**
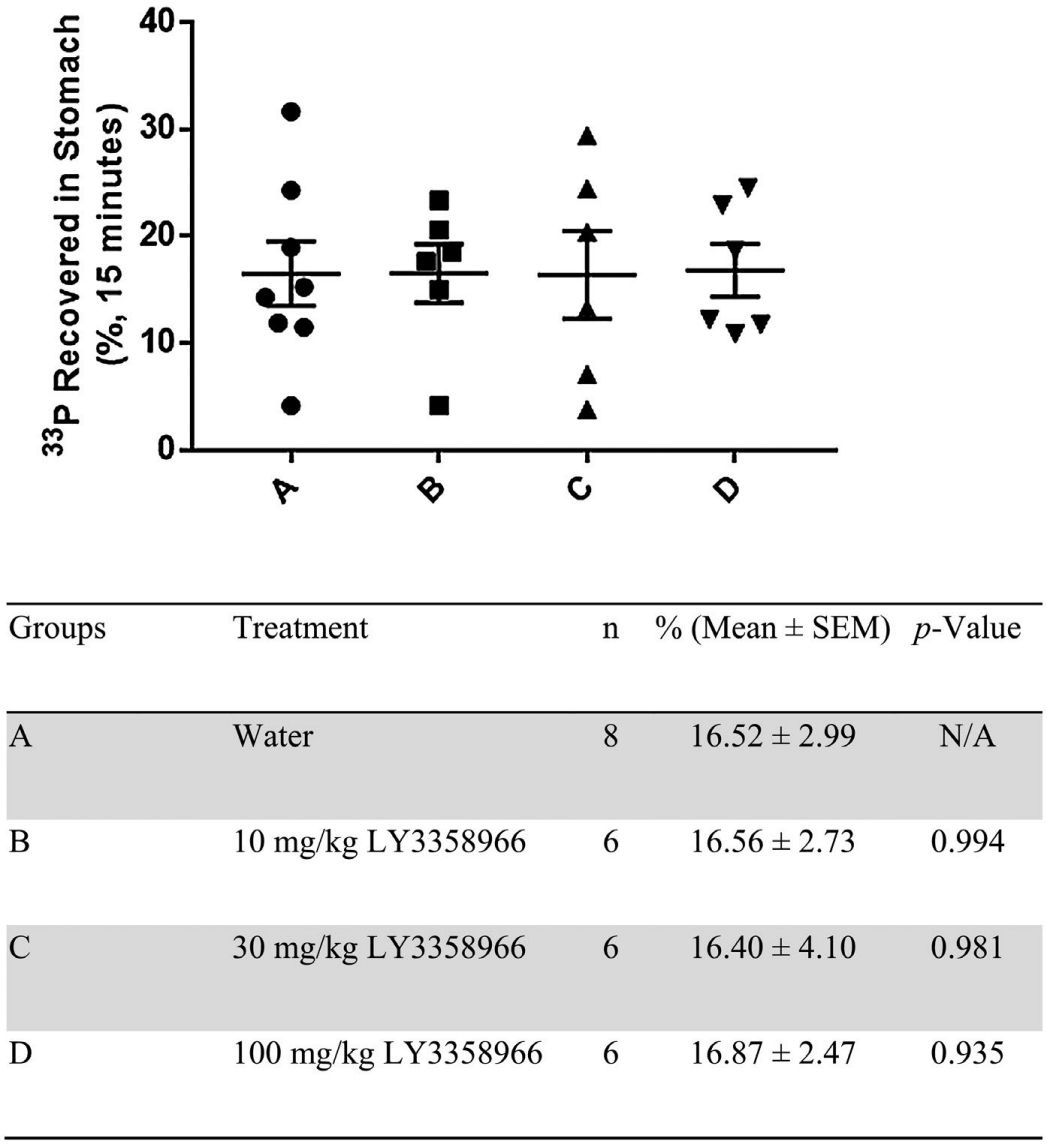
Effect of LY3358966 spray‐dried solid dispersion on gastric emptying in mice. Percentage of ^33^P‐phosphate retained in the stomach was defined as the percentage of administered dpm retained in the stomach at 30 min post the compound dose (15 min post ^33^P‐phosphate dose) and was presented as mean ± *SEM* with animal numbers equal to 8 for a water vehicle and 6 for LY3358966 treated groups. The *p*‐values were calculated using unpaired two‐tailed Student's *t*‐tests versus water control to optimize the chance of being significant

To confirm that the decrease in ^33^P‐phosphate appearing in the plasma of LY3358966‐treated animals is due to inhibition of ^33^P‐acute phosphate uptake, and not retention of ^33^P‐phosphate in the stomach, LY3358966 SDD was tested for its effect on gastric emptying in mice. LY3358966 at doses up to 100 mg/kg API had no effect on gastric emptying (Figure 4). This is an important finding because numerous compounds in our medicinal chemistry campaign inhibited gastric emptying and thus, were not pursued.

### 3.5 In vivo effect of LY3358966 SDD on phosphate absorption in mice when dosed as a solid in a capsule

The solubility and formulation of LY335896 are critical for its in vivo activity. A solid dose formulation of LY3358966, which is preferred as a human therapy, was developed. In the case of an NPT2b inhibitor, this necessitates that the solid formulation is solubilized in the stomach, at a pH where LY3358966 has minimal solubility, and remain in solution long enough to inhibit intestinal NPT2b. The ability of a solid SDD of LY3358966 to inhibit intestinal NPT2b was investigated in mice. The solid LY3358966 SDD formulation dosed in a capsule inhibited phosphate absorption (Table 3) comparably to LY3358966 SDD dosed in a solution (*p* = .46).

**TABLE 3:** Effect of LY3358966 spray‐dried solid dispersion on acute phosphate uptake into plasma in mice when dosed as a solid in a capsule or solution

Treatment	N	Dose mg/kg (API)	% Inhibition		*p*‐value^a^
			Mean	*SEM*	
Vehicle in solution	8	0	0	14.9	N/A
LY3358966 in solution	8	1.8	52.5	6.20	N/A
Vehicle in a capsule	10	0	0	15.5	N/A
LY3358966 in a capsule	10	1.8	58.5	5.13	.46

^a^ *p*‐value was calculated using a Student's *t*‐test with two tails to compare the percent inhibition by LY3358966 (mean ± *SEM*) dosed in a capsule versus in a solution.

### 3.6 The recovery of radiolabeled phosphate in feces of mice treated with LY3358966 SDD

**FIGURE 5:**
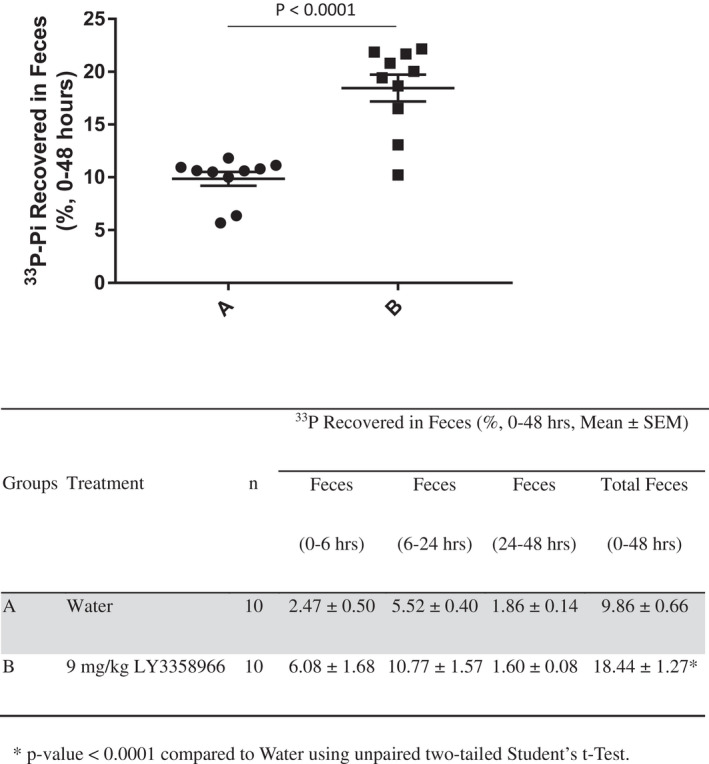
The recovery of radiolabeled phosphate in feces of mice treated with LY3358966 spray‐dried solid dispersion

To explore the ability of LY3358966 to block radiolabeled phosphate absorption longitudinally in mice, three doses of 9 mg/kg API of LY3358966 SDD were administered orally at 4‐h intervals. This dose was selected because it is about 8 times higher than the ED_50_ at 4 h post‐dosing LY3358966, and thus each dose should provide near‐complete inhibition of NPT2b for 4 h. Fifteen minutes after the second LY3358966 dose, a solution containing ^33^P‐phosphate was given to the animals. The feces were collected over 48 h and the ^33^P radioactivity was measured. Most of the ^33^P‐phosphate recovered in feces was within the first 24 h. In the placebo control group, ^33^P‐phosphate dosed in a solution was readily absorbed by the intestine and only 9.86% of the ^33^P‐phosphate dose was recovered in feces collected over 48 h (Figure 5). LY3358966 significantly increased the ^33^P‐phosphate recovered in the feces to 18.44% of the ^33^P‐phosphate dose (*p* < .0001, Figure 5), nearly doubling the percentage of ^33^P‐phosphate excreted in feces. Thus, LY3358966 inhibits phosphate absorption in mice.

### 3.7 The recovery of radiolabeled phosphate in feces or gastrointestinal tract of rats treated with LY3358966 SDD in the presence and absence of the phosphate binder, sevelamer

In rats, there is a modest effect of NPT2b inhibition on acute phosphate uptake into plasma, suggesting passive diffusion and/or other active transporters play a dominant role. This model was used to explore the ability of LY3358966, the phosphate binder, sevelamer, and these two in combination to inhibit phosphate absorption. Two complementary study designs were used. In both cases, rats were treated with a single dose of LY3358966 and/or sevelamer, 15 min prior to dosing a solution of ^33^P‐phosphate.

**FIGURE 6:**
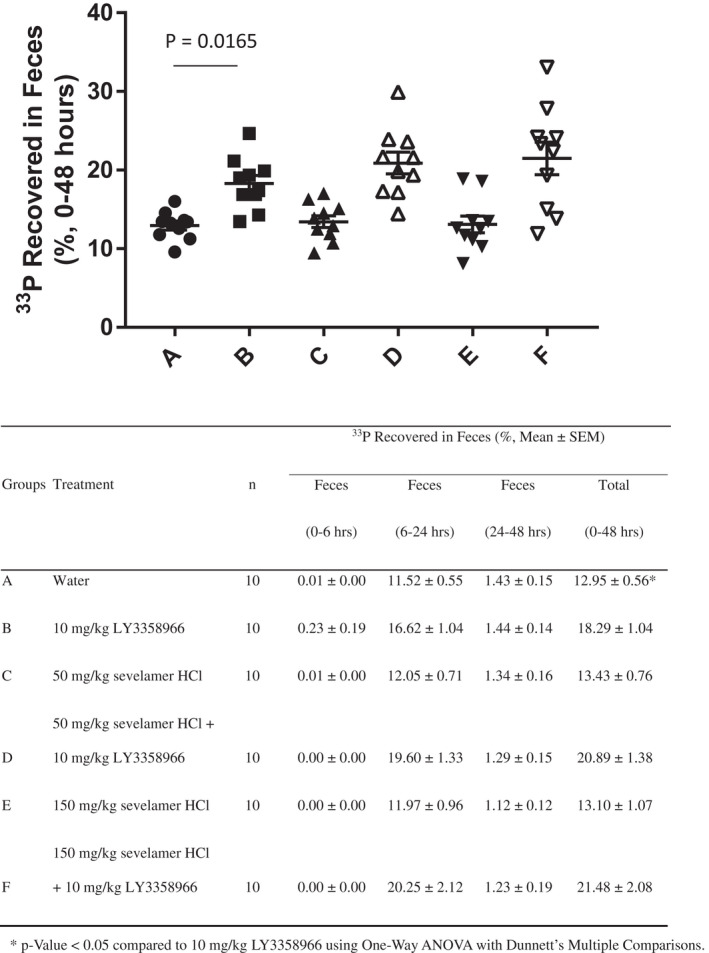
The recovery of radiolabeled phosphate in feces of rats treated with LY3358966 spray‐dried solid dispersion (SDD), sevelamer or an LY3358966 SDD/sevelamer combination

The first approach to assess the effect of LY3358966 on intestinal phosphate absorption in rats was to determine ^33^P‐phosphate excretion in feces collected for 48 h after dosing ^33^P‐phosphate, analogous to the mouse study. The 48‐h fecal recovery of ^33^P‐phosphate in the placebo‐treated group was 12.95% of the dose (Figure 6). The NPT2b inhibitor mediated recovery (difference between placebo and LY3358966‐treated groups) was 5.34% (*p* = .0165). Fifty and 150 mg/kg sevelamer did not increase ^33^P‐phosphate recovered from the 48‐h feces collection (13.43% and 13.10%, respectively, vs. 12.95% in the placebo group, Figure 6). When 50 or 150 mg/kg sevelamer was combined with LY3358966, there was a very modest numerical, but non‐significant, trend toward an increase in phosphate recovered (20.89% and 21.48%, respectively, versus 18.28% for the LY3358966 group, Figure 6).

**FIGURE 7:**
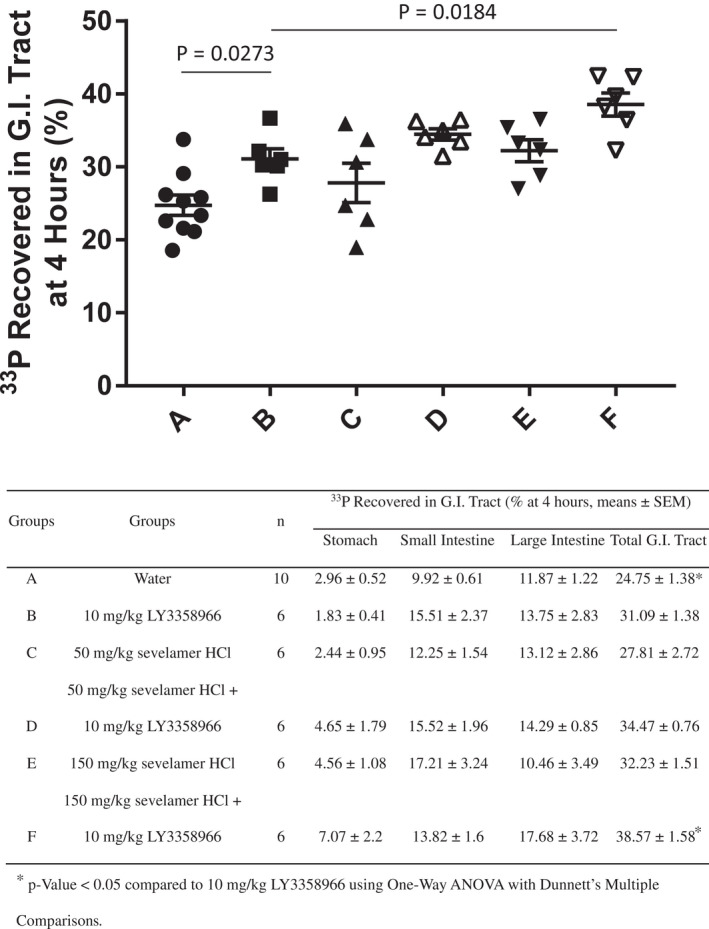
The recovery of radiolabeled phosphate in gastrointestinal (G.I.) tract of rats Treated with LY3358966 spray‐dried solid dispersion (SDD), sevelamer, or an LY3358966 SDD/sevelamer combination

In the second approach, feces and different sections of the rat gastrointestinal tract were collected 4 h after dosing ^33^P‐phosphate to measure the radioactive phosphate recovered in these sections. Since negligible ^33^P‐phosphate was recovered in feces over this time frame (see Figure 6 for an example), the feces were not further analyzed. The difference in the recovery of ^33^P‐phosphate between compound and placebo‐treated animals demonstrated the retention of radioactive tracer in the small and large intestines due to compound‐mediated inhibition of phosphate absorption. A 4‐h time point was selected because the vast majority of phosphate is absorbed within 4 h in humans^26^, ^27^ and thus would be a clinically relevant timepoint. In the vehicle‐treated group, 24.75% of the ^33^P‐phosphate dose was recovered, while in the 50 and 150 mg/kg sevelamer‐treated groups, ^33^P‐phosphate recovery was 27.81% and 32.23%, respectively (Figure 7). A 450 mg/kg dose of sevelamer was also tested, but that dose severely inhibited gastric emptying and was not further studied. LY3358966 at 10 mg/kg, significantly increased ^33^P‐phosphate recovery in the gastrointestinal tract 6.34% more than vehicle to 31.09% (*p* = .0273, Figure 7). The 6.34% recovery is similar to the 5.34% recovered in the 48‐h fecal recovery experiment (Figure 6). When 50 or 150 mg/kg sevelamer was combined with 10 mg/kg LY3358966, there was a further increase in ^33^P‐phosphate recovered from the gastrointestinal tract to 34.47% and 38.57%, respectively (Figure 7). The 150 mg/kg dose of sevelamer combined with LY3358966 significantly increased the recovery compared to the 31.09% recovery with LY3358966 alone (*p* = .0184). In combination, LY3358966 and sevelamer inhibited phosphate absorption 13.82% in this rat model.

## 4 DISCUSSION

The impaired ability of the kidney to excrete phosphate in CKD and ESRD has pathological consequences, leading to bone, kidney, and heart diseases. To lessen the burden of excess phosphate, different therapies to reduce the absorption of dietary phosphate in the intestine have been developed or proposed. These therapies either decrease the paracellular passive diffusion or transporter‐mediated active uptake of phosphate. In many cases, current therapies are inadequate, creating a need to find more effective therapies or combination therapies.

Previous ex vivo studies performed with genetic mouse models have indicated that the sodium phosphate cotransporter NPT2b dominates active transport in the mouse intestine.^19^, ^20^ However, if NPT2b is genetically deleted, the mouse still maintains its ability to absorb most of its bioavailable phosphate under normal dietary conditions, as the dietary phosphate recovered in feces only increases modestly with NPT2b ablation.^19^, ^28^, ^29^, ^30^ This highlights that in mice, there are redundant pathways for intestinal phosphate absorption. There is conflicting data in literatures on the importance of sodium‐dependent phosphate absorption in rats.^31^, ^32^

Unlike mice where the majority of NPT2b mRNA and protein expression is found in the ileum, in rats, the majority is found in the jejunum, with some found in the duodenum.^33^, ^34^ The rat NPT2b distribution is consistent with the more rapid absorption of phosphate found in human jejunum, compared to the ileum.^35^ A role for PiT1, PiT2, and/or another yet to be defined transporter has been proposed for rat intestinal phosphate absorption.^22^, ^23^, ^36^, ^37^ In rats, PiT1 mRNA is found in all three segments of the small intestine, but protein is mostly found in the jejunum with some in the duodenum. Low levels of PiT2 mRNA are expressed in all three segments of the small intestine. PiT2 protein is also found in all three segments.^33^, ^36^ In mice, both PiT1 and PiT2 mRNA is similarly expressed in the duodenum, jejunum, and ileum.^38^ Unless characterized in detail, transporter expression can be misleading in that expression levels are regulated in response to numerous factors (phosphate levels or 1,25‐dihydroxyvitamin D status) and the protein may be expressed on the basolateral membrane of the enterocyte or other cell types not involved in the absorption of phosphate from the intestinal lumen.

In this study, a potent NPT2b inhibitor, LY3358966, was used as a tool to explore the role of NPT2b mediated active transport in intestinal phosphate absorption. In vitro, LY3358966 is highly selective for NPT2b versus PiT1 and PiT2; however, it also inhibits NPT2a and NPT2c that are expressed in the kidney. However, LY3358966 has low bioavailability, and very high protein binding, thus it does not achieve an in vivo free fraction that would approach a concentration that could inhibit either of these transporters in our animal models. A pharmacological approach to study the role of NPT2b allows one to do short‐term studies which are less subject to compensatory regulation of competing absorption pathways that may occur in genetic models of NPT2b depletion. For example, proteins that may be involved in diffusional or paracellular phosphate transport pathways have been found to be significantly decreased in NPT2b knockout mice.^28^ Unlike many previous studies where the absorption of dietary phosphate was measured, we studied the absorption of radiolabeled soluble inorganic phosphate. While dietary phosphate may have bioavailability as low as 50%–60%,^19^, ^29^ the bioavailability of inorganic phosphate should approach 100%, allowing a more precise measure of the pharmacological intervention.

LY3358966 acutely (measured 15 min after dosing) inhibits phosphate uptake into plasma with an *E*_max_ of 74% in mice. Four hours after dosing, the *E*_max_ is 79%. We chose to study 4 h after dosing LY3358966 because in humans, most of the phosphate absorbed in normal humans or 1,25‐dihydroxyvitamin D‐depleted ESRD patients occurs in the first 4 h after phosphate ingestion.^26^, ^27^ The ability of LY3358966 to inhibit phosphate absorption in mice was determined by measuring the recovery of orally dosed ^33^P‐phosphate in feces over 48 h. Using multiple doses of LY3358966 to achieve near‐complete pharmacological inhibition of NPT2b for up to 8 h after dosing ^33^P‐phosphate, radiolabeled phosphate recovered in feces was increased 8.58%, from 9.86% in the control to 18.44%. This is comparable to what Knöpfel et al.^29^ found for the effect of genetic depletion of NPT2b on dietary phosphate absorption, while it is somewhat less than what Hernando et al.^19^ found. These results indicate that LY3358966 mediated inhibition of NPT2b in mice very effectively reduces acute phosphate uptake into plasma, but that there are redundant mechanisms for absorption, and it is much less effective on inhibiting phosphate absorption over time.

LY3358966 was found to produce a modest 22% decrease in the acute phosphate uptake into plasma in rats. We further compared the ability of LY3358966 to inhibit phosphate absorption to that of the phosphate binder, sevelamer, and the ability of the two to work in combination. Sevelamer will decrease the free phosphate concentration in the intestinal lumen, and thus, decrease diffusional phosphate absorption. LY3358966 had a modest effect on ^33^P‐phosphate recovered in rat feces over 48 h, while sevelamer at both doses tested had no effect. The observed effects of LY3358966 or the binder may be anticipated from previous studies in rats which showed more rapid phosphate absorption rates in the proximal intestine, but nevertheless, an appreciable rate of absorption in other intestinal segments with slower transit times which would allow for substantial phosphate absorption over time.^39^, ^40^ To further explore this, we used a second model in which the recovery of ^33^P‐phosphate in the gastrointestinal tract of the rat 4 h after dosing phosphate was measured. We chose this time point to minimize the effects of prolonged, slow absorption of phosphate, which does not appear to occur in humans.^27^ In this study, there were again modest effects of LY3358966 on phosphate retained in the intestine at 4 h, consistent with those found in the 48‐h fecal recovery protocol (6.34% vs. 5.34% recovered, respectively). In this model, modest effects of sevelamer alone were also apparent and it appeared to work additively with LY3358966.

Our studies highlight the importance of careful characterization of the pharmacological agents studied in the preclinical model. Somewhat surprisingly, sevelamer at the doses tested, 50 and 150 mg/kg, had minimal effect on intestinal phosphate absorption. The doses tested would translate to 3.5 and 10.5 g doses for a 70 kg human. The sevelamer phosphate binding capacity exceeded the phosphate dose around threefold at the 50 mg/kg dose, and ninefold at the 150 mg/kg dose.^41^ The higher dose of sevelamer was found to produce profound inhibition of gastric emptying in our model. We also observed significant inhibition of gastric emptying with some compounds in the chemical series explored in our discovery campaign. Importantly, LY3358966 showed no evidence of any effect on gastric emptying at the doses tested. Others have also reported effects on gastrointestinal motor function with their active phosphate transporter inhibitors in preclinical models.^23^

There are some limitations to our studies. The methodology employed to measure acute uptake of radiolabeled phosphate into plasma assumes there is no difference in plasma clearance of phosphate between groups and that measuring plasma ^33^P‐phosphate at a single time point, rather than a full ^33^P absorption curve, adequately measures target engagement and inhibition of intestinal NPT2b, the primary goals for the study. Another weakness of our studies is that we never performed chronic dosing or studied LY335896 in disease models. Others have reported beneficial effects of therapies that limit the phosphate absorption in rodent CKD models.^42^, ^43^, ^44^, ^45^, ^46^ Using the clinically relevant doses employed in our studies, it is unlikely we would observe any benefits in a disease model, as we anticipate a little impact on the phosphate burden. Many studies that have shown benefit in preclinical renal disease models used very large doses of phosphate binders incorporated in the food (3%–5% of food or around 1.5 to 3.5 g/kg/day assuming standard rates of food consumption for rats and mice) that may produce effects on phosphate metabolism that cannot be achieved clinically or unanticipated side effects, for example, effects on food intake. Caloric intake is known to affect renal function in rodent CKD models.^47^, ^48^, ^49^

1,25‐dihydroxyvitamin D, which has been shown to upregulate intestinal NPT2b in preclinical models,^24^, ^25^, ^50^ also increases phosphate absorption^11^, ^51^ and transporter‐mediated phosphate absorption^16^ in ESRD patients, suggesting a role for NPT2b in human phosphate absorption. Nicotinamide therapy reduces hyperphosphatemia in patients on dialysis.^52^ Preclinical studies indicate the effect of nicotinamides is through decreased NPT2b mediated transport,^20^, ^53^ providing additional evidence for an appreciable role of NPT2b in human phosphate absorption. Two NPT2b inhibitors that were reported to decrease phosphate absorption in preclinical models recently advanced to the clinic. While one compound, ASP3325, had no apparent effect on phosphate absorption in the population studied,^54^ a second, DS‐2330b, may have had a small effect that was dependent upon the solubility of the compound in the intestinal lumen.^55^ The preclinical profiles of these two compounds have not been published to date, so the translation between their preclinical models and the clinical cannot be assessed. A nonselective pan NPT2b, PiT1, and PiT2 inhibitor decreased phosphate absorption in rats^23^ and humans.^22^ These preclinical and human studies highlight the need for well‐characterized tools that can be used to further elucidate the roles of the different active and passive pathways responsible for intestinal phosphate absorption under various conditions. Importantly, these tools can be used to identify combination therapies that can be used to inhibit redundant phosphate absorption pathways, and effectively address the high phosphate burden in patients with CKD and ESRD.

## DISCLOSURE

XW, XY, AD, HYZ, CMZ, GP, WFM, LP, JAP, BEM, and JVH are current Eli Lilly employees. YX, DW, CAR, MCK, and JRW are Eli Lilly retirees.

## ETHICAL APPROVAL

All animal procedures were approved by and conducted in accordance with the Eli Lilly and Covance Institutional Animal Care and Use Committee guidelines.

## AUTHOR CONTRIBUTIONS

Wang, Xu, Yu, Dey, Zhang, Wodka, Peterson, Mattioni, Kowala, Haas, and Wetterau designed the studies; Xu, Wodka, and Peterson designed, synthesized, and formulated LY3358966; Wodka, Porras, Peterson, Zink, Zhang, Dey, Porter, Matter, Reidy, and Mattioni carried out experiments; Wang, Yu, Xu, Wodka, Porras, Dey, Zink, Mattioni, Haas, and Wetterau analyzed the data; Wang, Yu, Zink, and Dey made the tables and figures; Wang, Yu, Kowala, and Wetterau drafted and revised the paper; and all authors approved the final version of the manuscript.

## Supporting information

Supplementary Material

## Data Availability

Data and protocol will be made available upon request.
