# Supplementary material for: Effects of pharmacological inhibition of the sodium‐dependent phosphate cotransporter 2b (NPT2b) on intestinal phosphate absorption in mouse and rat models
Source: Pharmacol Res Perspect. 2022 Feb 22;10(2):e00938. doi: 10.1002/prp2.938 (PMC8863579; doi:10.1002/prp2.938)
Supplement: Supplementary file 1 — Supplementary Material [file PRP2-10-e00938-s001.zip › prp2938-sup-0001-FigS1.docx]

**Effects of Pharmacological Inhibition of the Sodium Dependent Phosphate Cotransporter 2b (NPT2b) on Intestinal Phosphate Absorption in Mouse and Rat Models**

Xiaojun Wang*, Yanping Xu, Xiaohong Yu, Asim Dey, Hong Y. Zhang, Charity M. Zink, Derek Wodka, Regina Porter, William F. Matter, Leah Porras, Charles A. Reidy, Jeffrey A. Peterson, Brian E. Mattioni, Joseph V. Haas, Mark C. Kowala, and John R. Wetterau

Lilly Research Laboratories, Eli Lilly and Company, Indianapolis, IN 46285, USA

**Journal of Pharmacology and Experimental Therapeutic**

**Scheme 1 : Synthesis of LY3359866**
