# Supplementary material for: Effects of pharmacological inhibition of the sodium‐dependent phosphate cotransporter 2b (NPT2b) on intestinal phosphate absorption in mouse and rat models
Source: Pharmacol Res Perspect. 2022 Feb 22;10(2):e00938. doi: 10.1002/prp2.938 (PMC8863579; doi:10.1002/prp2.938)
Supplement: Supplementary file 1 — Supplementary Material [file PRP2-10-e00938-s001.zip › prp2938-sup-0002-Text.docx]

**Effects of Pharmacological Inhibition of the Sodium Dependent Phosphate Cotransporter 2b (NPT2b) on Intestinal Phosphate Absorption in Mouse and Rat Models**

Xiaojun Wang*, Yanping Xu, Xiaohong Yu, Asim Dey, Hong Y. Zhang, Charity M. Zink, Derek Wodka, Regina Porter, William F. Matter, Leah Porras, Charles A. Reidy, Jeffrey A. Peterson, Brian E. Mattioni, Joseph V. Haas, Mark C. Kowala, and John R. Wetterau

Lilly Research Laboratories, Eli Lilly and Company, Indianapolis, IN 46285, USA

**Journal of Pharmacology and Experimental Therapeutic**

**Supplemental Methods**

All reagents were purchased from commercial vendors and used without any further manipulation. LC-ES/MS analysis was performed on an Agilent HP1100 liquid chromatography system. Electrospray mass spectrometry measurements (acquired in positive and/or negative mode) were performed on a Mass Selective Detector quadrupole mass spectrometer that was interfaced to the HP1100 HPLC. NMR spectra were obtained on a Bruker AVIII HD 400 MHz NMR Spectrometer or a Varian VNMRS 300 or 400 MHz NMR Spectrometer. Chemical shifts are reported in parts per million and referenced to CDCl_3_ (7.26 ppm for ^1^H) or DMSO-d_6_ (2.50 ppm for ^1^H) as internal standard. Peak multiplicities are reported as s (singlet), d (doublet), t (triplet), q (quartet), m (multiplet), br s (broad singlet), dd (doublet of doublets) and dt (doublet of triplets). Coupling constants (*J*) are reported in hertz (Hz).

## Full-Length cDNA Cloning and Generation of Cell Lines Over-Expressing Human, Mouse, and Rat NPT2b

The vendors for target cDNA are listed in supplemental Table 1. Their sequences were verified by full-length sequencing. The target cDNA was cloned into pcDNA5/TO for mammalian expression using T-Rex System^TM^ (ThermoFisher Scientific) and was then stably transfected into CHO-K1 cells using standard mammalian transfection procedures. NPT2b over-expressed in CHO-TREX cells upon induced by tetracycline (Sigma). A subclone from each over-expressing cell line was selected based on resistance to hygromycin and blasticidin (ThermoFisher Scientific) and activity in an assay measuring the sodium-dependent uptake of ^33^P-Pi described in Method section. Stable over-expressing cells were maintained using standard cell culture techniques.

## Full Length cDNA Cloning and Generation of Cell Lines Over-Expressing Human NPT2a, NPT2c, Pit1, and Pit2

The vendors for target cDNA are listed in Supplemental Table 2. Their sequences were verified by full-length sequencing. The cDNA coding human NPT2a or NPT2c was cloned into pJTI R4 CMV-TO for mammalian expression using T-Rex System^TM^ (ThermoFisher Scientific). The cDNA coding human Pit1 and Pit2 was cloned into pcDNA3.1(+) for mammalian constitutive expression. The appropriate cDNA was then stably transfected into CHO-K1 cells using standard mammalian transfection procedures. Human NPT2a and NPT2c over-expressed in CHO-TREX cells upon induced by tetracycline (Sigma). Human Pit1 and Pit2 over-expressed in CHO cells constitutively. A subclone from each over-expressing cell line was selected based on resistance to hygromycin and blasticidin (ThermoFisher Scientific) for NPT2a and NPT2c, or to Geneticin for Pit1 and Pit2, and activity in an assay measuring the sodium-dependent uptake of ^33^P-Pi for NPT2a, NPT2c, Pit1, and Pit2 as described in Method section. Stable over-expressing cells were maintained using standard cell culture techniques.

**Supplemental Table 1: Summary of cDNA Used to Over-Express NPT2b in CHO-TREX Cells**

| Gene | Species | Accession Number | Vendor |
| --- | --- | --- | --- |
| NPT2b (slc34A2) | Human | NM_006424.2 | Openbiosystems Co. (Cat#: MHS1010-99823026, Clone ID: 40148124) |
| NPT2b (slc34A2) | Mouse | NM_011402.3 | Openbiosystems Co. (Cat#: MMM1013-98478398, Clone ID: 3962784) |
| NPT2b (slc34A2) | Rat | NM_053380.2 | Openbiosystems Co. (Cat#: MRN1768-9510282, Clone ID: 7099656) |

**Supplemental Table 2: Summary of cDNA Used to Over-Express Other Sodium Dependent Transporters**

| Gene | Species | Accession number | Vendor |
| --- | --- | --- | --- |
| NPT2a (slc34A1) | Human | NM_003052.4 | Openbiosystems Co. (Cat#: MHS1010-9205591, Clone ID: 5186103) |
| NPT2c (slc34A3) | Human | NM_080877.2 | Synthesize at GeneOracle |
| Pit1 (slc20A1) | Human | NM_005415.4 | OriGene Technologies, Inc. (SKU: SC322456) |
| Pit2 (slc20A2) | Human | NM_006749.3 | Openbiosystems Co. (Cat#: MHS1010-7295771, Clone ID: 4820601) |

**Synthesis of LY3359866**

**tert-Butyl 4-[(4-methoxy-4-oxo-butyl)carbamoyl]-2,2-dimethyl-piperazine-1-carboxylate (2)**. A solution of t-butyl 2,2-dimethylpiperazine-1-carboxylate (500 mg, 2.28 mmol) in DCM (12 mL) was cooled in an ice/water bath and diisopropylethylamine (1.20 mL, 6.85 mmol) was added in one portion. A solution of methyl 4-isocyanatobutanoate (447 mg, 2.97 mmol) in DCM (3 mL) was added drop wise over 5 minutes and the reaction mixture was slowly warmed to RT and stirred for an additional 15 minutes. The reaction mixture was then partitioned between 5% aqueous citric acid (100 mL) and DCM (20 mL). The organic layer was separated, and the aqueous layer was extracted with DCM (2 x 20 mL). The combined organic extracts were washed sequentially with saturated aqueous NaHCO_3_ (30 mL), brine (30 mL), dried over anhydrous Na_2_SO_4_, filtered, and concentrated under reduced pressure. The resulting residue was purified by silica chromatography (30-50% hexanes/acetone) to obtain the title compound as colorless viscous oil (848 mg, 95%). ^1^H NMR (400 MHz, CDCl_3_) d 1.34 (s, 6H), 1.45 (s, 9H), 1.83 (t, *J* = 6.9 Hz, 2H), 2.37 (t, *J* = 7.1 Hz, 2H), 3.25-3.30 (m, 2H), 3.37 (t, *J* = 5.7 Hz, 2H), 3.47 (s, 2H), 3.65 (s, 3H), 3.71 (t, *J* = 5.7 Hz, 2H), 4.65-4.68 (m, 1H). LC-ES/MS (m/z) 358 [M+H]^+^.

**tert-butyl 4-(4 -hydroxybutylcarbamoyl)-2,2-dimethyl-piperazine-1-carboxylate (3)**. To a solution of tert-butyl 4-[(4-methoxy-4-oxo-butyl)carbamoyl]-2,2-dimethyl-piperazine-1-carboxylate (783 mg, 2.01 mmol) in THF (2 mL) at room temperature was added a 2M solution of LiBH_4_ in THF (3.02 mL, 6.04 mmol) drop wise and the resulting mixture was stirred at room temperature for 12 hours. The reaction mixture was quenched with 0.5 mL of MeOH, stirred at room temperature for 20 minutes, and partitioned between 5% aqueous NaHCO_3_ (150 mL) and DCM (50 mL). The organic layer was separated, and the aqueous layer was extracted twice with DCM (2 x 50 mL). The combined organic layers were washed with brine (50 mL), dried over anhydrous Na_2_SO_4_, filtered, and evaporated to dryness under reduced pressure to afford the title compound as a white solid (634 mg, 96%).  ^1^H NMR (400 MHz, CDCl_3_) d 1.36 (s, 6H), 1.46 (s, 9H), 1.58-1.63 (m, 4H), 3.31-3.28 (m, 2H), 3.49 (s, 2H), 3.66-3.69 (m, 2H), 3.71-3.74 (m, 2H), 3.37-3.39 (m, 2H). LC-ES/MS (m/z) 330 [M+H]^+^.

**N-(4 -hydroxybutyl)-3,3-dimethyl-piperazine-1-carboxamide hydrochloride (4).** To a solution of tert-butyl 4-(4- hydroxybutylcarbamoyl)-2,2-dimethyl-piperazine-1-carboxylate (631 mg, 1.92 mmol) in DCM (20 mL) was added 4N HCl in dioxane (2.4 mL, 9.58 mmol) drop wise over 5 minutes and the resulting solution was stirred at room temperature for 2 hours. The volatiles were removed in vacuo and the residue was dried under vacuum to afford the title compound as a hygroscopic white oily solid (quantitative), suitable for use in the next step without further purification. LC-ES/MS (m/z) 230 [M+H]^+^.

**Methyl 4-[2-(4-amino-2,6-difluoro-phenyl)ethynyl]benzoate (5)**. A suspension of 3,5-difluoro-4-iodoaniline (14.7 g, 55.9 mmol), Cul (0.745 g, 3.91 mmol), bis(triphenylphosphine)palladium (II) dichloride (1.59 g, 2.24 mmol), methyl 4- ethynylbenzoate (9.05 g, 55.9 mmol), triethylamine (114 mL) in THF (44.1 mL) was stirred at 60^o^C for 3 hours. After cooling to room temperature, the solvent was evaporated to dryness under reduced pressure. Ethyl acetate (100 mL) and water (100 mL) were added, and the resulting solid was filtered over diatomaceous earth. The organic layer from the filtrate was separated, dried over anhydrous MgSO4, and evaporated to dryness under reduced pressure. A 1:1 mixture of dichloromethane:heptane (400 mL) was added to the resulting residue and the mixture was stirred at room temperature overnight. The resulting solid was collected by filtration and dried under vacuum to afford the title compound (8.0 g, 46%) as a brown solid. ^1^H NMR (300 MHz, DMSO-d_6_) d 3.86 (s, 3H), 6.26-6.37 (m, 4H), 7.59 (d, *J* = 8.2 Hz, 2H), 7.96 (d, *J* = 8.5 Hz, 2H).

**Methyl 4-[2-(4-amino-2,6-difluoro-phenyl)ethyl]benzoate (6)**. A mixture of methyl 4-[2-(4-amino-2,6-difluoro-phenyl)ethynyl]benzoate (1.17 g, 3.38 mmol) and palladium black (0.53 g, 5.0 mmol) in a 4:1 solution of methanol/THF (50 mL) was hydrogenated at 60 psi at 40^o^C for 14 hours using a Parr shaker. The resulting suspension was filtered through a pad of diatomaceous earth and evaporated to dryness in vacuo. The resulting residue was purified by silica chromatography (25-35% hexanes/THF gradient) to afford the title compound as a white solid (404 mg, 40%). ^1^H NMR (400 MHz, DMSO-d_6_) d 2.71-2.83 (m, 4H), 3.84 (s, 3H), 5.52 (s, 2H), 6.10-6.15 (m, 2H), 7.28 (d, *J* = 8.3 Hz, 2H), 7.85 (d, *J* = 8.2 Hz, 2H). LC-ES/MS (m/z) 292 [M+H]^+^.

**Methyl 4-[2-[4-[[2-(tert-butoxvcarbonylamino)-4,5,6,7-tetrahydrobenzothiophene-3- carbonyl]amino]-2,6-difluoro-phenyil]ethyl]benzoate (7)**. To a cooled suspension of methyl 4-[2-(4-amino-2,6-difluoro-phenyl)ethyl]benzoate (2.11 g, 7.24 mmol) and 3-(tert-butoxycarbonylamino)-4,5,6,7-tetrahydrobenzothiophene-2-carboxylic acid (237 g, 7.97 mmol) in DCM (60 mL) was added diisopropylethylamine (5.05 mL, 29.0 mmol). Bis-(2-oxo-3-oxazolidinyl)phosphinic chloride (2.30 g, 9.05 mmol) was then added in small portions over 30 minutes at 0^o^C and the reaction mixture was then warmed to RT and stirred for 24 hours. Additional 3-(tert-butoxycarbonylamino)-4,5,6,7-tetrahydrobenzothiophene-2-carboxylic acid (0.6 g, 2.0 mmol) was added, followed by diisopropylethylamine (1.26 mL, 7.25 mmol) and bis-(2-oxo-3-oxazolidinyl)phosphinic chloride (0.58 g, 2.26 mmol) in small portions over 5 minutes. The resulting turbid brown reaction mixture was stirred at room temperature for 12 hours, partitioned between 5% aqueous citric acid (150 mL) and DCM (25 mL), and the organic layer was separated. The aqueous layer was extracted with DCM (2 x 50 mL). The combined organic extracts were washed sequentially with 10% aqueous NaHCO_3_ (50 mL), brine (50 mL), dried over anhydrous Na_2_SO_4_, filtered and concentrated under reduced pressure. The resulting brown oily solid was purified by silica gel chromatography (15-40% hexanes/10% methyl tert-butyl ether in DCM) to afford desired product, which was triturated with methyl tert-butyl ether (15 mL). The resulting solid was collected by filtration and dried under vacuum to afford the title compound (2.75 g, 66%). ^1^H NMR (400 MHz, DMSO-d_6_) d 1.44 (s, 9H), 1.69-1.77 (m, 4H), 2.53-2.65 (m, 4H), 2.91 (br s, 4H), 3.84 (s, 3H), 7.30-7.34 (m, 4H), 7.86 (d, *J* = 8.3 Hz, 2H), 9.80 (br s, 1H), 9.94 (s, 1H). LC-ES/MS (m/z) 569 [M-1].

**methyl 4-[2-[4-[(2-amino-4,5,6,7-tetrahydrobenzothiophene-3-carbonyl)amino]-2,6-difluoro-phenyl]ethyl]benzoate hydrochloride (8).** To a solution of methyl 4-[2-[4-[[2-(tert-butoxycarbonylamino)-4,5,6,7-tetrahydrobenzothiophene-3-carbonyl]amino]-2,6-difluoro-phenyl]ethyl]benzoate (351 mg, 0.61 mmol) in DCM (6 mL) under nitrogen was added 4N HCl in dioxane (155 ml, 6.1 mmol) was added drop wise over 5 minutes and the resulting solution was stirred at room temperature for 12 hours. The reaction mixture was concentrated in vacuo and the resulting pale yellow residue was triturated with a minimal amount of DCM. The resulting solid was collected by filtration and dried under vacuum to afford the title compound as an off-white powder (337 mg, 99%). ^1^H NMR (400 MHz, DMSO-d_6_) d 1.73-1.75 (m, 4H), 2.40-2.44 (m, 2H), 2.56-2.59 (m, 2H), 2.85 (s, 4H), 3.80 (s, 3H), 7.26-7.28 (m, 4H),7.81-7.83 (m, 2H), 9.21 (s, 1H). LC-ES/MS (m/z) 571 [M-1].

**ethyl 4-[2-[4-[[2-[[3-(chloromethvl)benzoyl]aminio]-4,5,6,7-tetrahydrobenzothiophene-3- carbonyl]amino]-2,6-ditIuoro-phenyl]ethyl]benzoate (9)**. To a cooled suspension of methyl 4-[2-[4-[(2-amino-4,5,6,7-tetrahydrobenzothiophene-3-carbonyl)amino]-2,6-difluoro-phenyl]ethyl)benzoate hydrochloride (2.43 g, 4.55 mmol) in DCM (80 mL) to 0^o^C with an ice/water bath was added pyridine (0.92 mL, 11 mmol) drop wise with stirring over 5 minutes. The resulting solution was stirred for an additional 5 min at 0^o^C and a solution of 3-(chloromethyl)benzoyl chloride (0.71 mL, 5.0 mmol) in DCM (20 mL) was added drop wise over 5 minutes. The reaction mixture was stirred for additional 30 min at 0^o^C, diluted with 10% aqueous citric acid (150 mL) and stirred at room temperature for 1 hour. The organic layer was separated, and the aqueous layer was extracted with DCM (2 x 50 mL). The combined organic extracts were washed sequentially with 5% aqueous NaHCO_3_ (2 x 50 mL), brine (50 mL), dried over anhydrous Na_2_SO_4_, filtered, and evaporated to dryness under reduced pressure. The resulting residue was triturated with ethanol (30 mL). The resulting solid was collected by filtration, washed with ethanol (15 mL), and dried under vacuum to afford the title compound as a tan solid (2.61 g, 92%). ^1^H NMR (400 MHz, DMSO-d_6_) d 1.73-1.81 (m, 4H), 2.68 (m, 4H), 2.92 (s, 4H), 3.84 (s, 3H), 4.83 (s, 2H), 7.32 (d, *J* = 8.3 Hz, 2H) 7.38-7.34 (m, 2H), 7.56 (t, *J* = 7.7 Hz, 1H), 7.69 (d, *J* = 7.8 Hz, 1H), 7.82 (d, *J* = 7.9 Hz, 1H), 7.87 (d, *J* = 8.2 Hz, 2H), 7.96 (br s, 1H), 10.10 (s, 1H), 11.34 (s, 1H). LC-ES/MS (m/z-) 621 [M-1].

**methyl 4-[2-[2,6-difluoro-4-[[2-[[3-[[4-(4-hydroxy butylcarbamoyl)-2, 2-dimethyl-piperazin-1-yl]imethyl]benzoyl]amino]-4,5,6,7-tetrahydrobenzothiophene-3 carbonyl]amino] phenyl]ethyl]benzoate (10).** A mixture of methyl 4-[2-[4-[[2-[[3-(chloromethyl)benzoyl]amino]-4,5,6,7-tetrahydrobenzothiophene-3-carbonyl]amino]-2,6- difluoro-phenyl]ethyl]benzoate (50 mg, 0.08 mmol), N-(4-hydroxybutyl)-3,3-dimethyl-piperazine-1-carboxamide hydrochloride (42.7 mg, 0.16 mmol) and diisopropylethylamine (0.056 mL, 0.32 mmol) in acetonitrile (1.5 mL) and methanol (50 mL) was microwaved at 110^o^C for 4 hours using a BIOTAGE® Initiator microwave synthesizer. The reaction mixture was concentrated in vacuo and the residue was partitioned between 5% aqueous NaHCO_3_ (75 mL) and DCM (25 mL). The organic layer was separated, and the aqueous layer was extracted with DCM (2 x 25 mL). The combined organic extracts were washed with brine (25 mL), dried over anhydrous Na_2_SO_4_, filtered, and evaporated to dryness under reduced pressure. The resulting residue was purified by reverse phase chromatography over C-18 silica, eluting with a gradient of 0-100% of a mixture of 5% HCOOH in H_2_O/ACN, to afford the title compound as a light yellow foamy solid (30.2 mg, 46%).  ^1^H NMR (400 MHz, DMSO-d_6_) d 1.05 (s, 6H), 1.37-1.44 (m, 4H), 1.80-1.85 (m, 4H), 2.25-2.27 (m, 2H), 2.65-2.67 (m, 4H), 2.91 (br s, 4H), 2.96-3.05 (m, 2H), 3.12 (s, 2H), 3.17-3.26 (m, 2H), 3.35-3.41 (m, 2H), 3.51 (s, 2H), 3.84 (s, 3H), 4.36 (t, *J* = 5.1 Hz, 1H), 6.35-6.39 (m, 1H), 7.32 (d, *J* = 8.3 Hz, 2H), 7.51-7.56 (m, 4H), 7.70-7.80 (m, 1H), 7.87 (m, 3H), 10.01 (br s, 1H), 11.45 (br s, 1H). LC-ES/MS (m/z) 816 [M+H]^+^.

**4-[2-[2,6-difluoro-4-[[2-[[3-[[4-(4-hydroxybutylcarbamoyl)-2,2-dimethyl-piperazin-1-yl]methyl]benzoyl]amino]-4, 5,6,7-tetrahydrobenzothiophene-3-carbonyl]amino]phenyl]ethyl]benzoic acid, LY3358966 (1)**. A suspension of methyl 4-[2-[2,6-difluoro-4-[[2-[[3-[[4-(4-hydroxybutylcarbamoyl)-2,2-dimethyl-piperazin-1-yl]methyl]benzoyl]amino]-4,5,6,7-tetrahydrobenzothiophene-3-carbonyl]amino]phenyl]ethyl]benzoate (196 mg, 0.24 mmol) and lithium hydroxide monohydrate (17.2 mg, 0.72 mmol) in THF (4 mL)/MeOH (2 mL) and water (2 mL) was stirred at room temperature for 12 hours. The reaction mixture was then diluted with 4 mL of water and concentrated under reduced pressure to approximately half of the volume. An aqueous solution of 1N HCI was added drop wise to provide a thick off-white suspension, which was evaporated to dryness in vacuo. The resulting residue was purified by reverse phase chromatography over C-18 silica, eluting with a gradient of 0-100% of a mixture of 5% ammonium formate in H_2_O/ACN, to afford the title compound as a pale yellow solid (82.5 mg, 41%). ^1^H NMR (400 MHz, DMSO-d_6_) d 1.05 (s, 6H), 1.37-1.42 (m, 4H), 1.74-1.85 (m, 4H), 2.25-2.30 (m, 2H), 2.64-2.76 (m, 4H), 2.91 (s, 4H), 2.96-3.02 (m, 2H), 3.12-3.15 (m, 2H), 3.17-3.25 (m, 2H), 3.35-3.40 (m, 2H), 3.51 (s, 2H), 4.36 (t, *J* = 5.1 Hz, 1H), 6.36 (t, *J* = 5.4 Hz, 1H), 7.30 (d, *J* = 8.3 Hz, 2H), 7.45-7.55 (m, 4H), 7.74-7.79 (m, 1H), 7.84-7.87 (m, 4H), 10.01 (s, 1H), 11.45 (s, 1H), 12.82 (br s, 1H). LC-ES/MS (m/z) 802 [M+H]^+^.
